# Peptide-Carbon Quantum Dots conjugate, Derived from Human Retinoic Acid Receptor Responder Protein 2, against Antibiotic-Resistant Gram Positive and Gram Negative Pathogenic Bacteria

**DOI:** 10.3390/nano10020325

**Published:** 2020-02-14

**Authors:** Aninda Mazumdar, Yazan Haddad, Vedran Milosavljevic, Hana Michalkova, Roman Guran, Sukanya Bhowmick, Amitava Moulick

**Affiliations:** 1 Department of Chemistry and Biochemistry, Mendel University in Brno, Zemedelska 1, CZ-613 00 Brno, Czech Republic; yazanhaddad@hotmail.com (Y.H.); grizlidripac@gmail.com (V.M.); hanabuchtelova@gmail.com (H.M.); r.guran@email.cz (R.G.); sukanyabhowmick1992@gmail.com (S.B.); 2 Central European Institute of Technology, Brno University of Technology, Purkynova 123, CZ-612 00 Brno, Czech Republic

**Keywords:** bacterial infections, antibiotic-resistant, carbon quantum dots, antibacterial activity, toxicity

## Abstract

Antibiotic-resistant bacterial infections have become global issues for public health, which increases the utter need to develop alternatives to antibiotics. Here, the HSER (*Homo sapiens* retinoic acid receptor) peptide was designed from retinoic acid receptor responder protein 2 of *Homo sapiens,* and was conjugated with synthesized CQDs (carbon quantum dots) for enhanced antibacterial activity in combination, as individually they are not highly effective. The HSER–CQDs were characterized using spectrophotometer, HPLC coupled with electrospray-ionization quadrupole time-of-flight mass spectrometer (ESI–qTOF) mass spectrometer, zeta potential, zeta size, and FTIR. Thereafter, the antibacterial activity against Vancomycin-Resistant *Staphylococcus aureus* (VRSA) and *Escherichia coli* (carbapenem resistant) was studied using growth curve analysis, further supported by microscopic images showing the presence of cell debris and dead bacterial cells. The antibacterial mechanism of HSER–CQDs was observed to be via cell wall disruption and also interaction with gDNA (genomic DNA). Finally, toxicity test against normal human epithelial cells showed no toxicity, confirmed by microscopic analysis. Thus, the HSER–CQDs conjugate, having high stability and low toxicity with prominent antibacterial activity, can be used as a potential antibacterial agent.

## 1. Introduction

The evolution of bacteria with every generation to acquire resistance towards new antibiotics have made it a challenge to treat pathogenic bacteria, as their pattern of resistance differs [1]. Thus, bacterial infections due to the rapid increase in antibiotic resistance have become a major threat to global health. Antibiotic resistance occurs due to incomplete course of antibiotic dose and its misuse [2], exposure to constant stress, changes in genomic level, and horizontal gene transfer. The number of multidrug-resistant pathogenic bacteria is increasing every day, creating worse situations for the clinical settings to deal with it [3,4].

The World Health Organization in the year 2017 listed the antibiotic-resistant bacteria in three different categories depending on the threat level priorities: critical, high, and medium [5]. The Vancomycin-Resistant *Staphylococcus aureus* (VRSA) is at the highest threat level and the carbapenem-resistant *Enterobacteriaceae* (CRE) is in the critical level. The last resort for the gram positive and negative bacteria is vancomycin and carbapenem, but these are also becoming resistant, creating global epidemiological and clinical burden. The VRSA is resistant due to its ability to reject the entry of vancomycin into its own system and prevent its own altered autolysis, along with gene transcription alteration [6]. Whereas, the Enterobacteriaceae are resistant due to the development of carbapenemase [7,8]. Antimicrobial agents like fosfomycin, polymyxin, colistin, and nitrofurantoin have become ineffective due to plasmid-mediated resistance in CRE. Whereas combinatorial treatment using different antibiotics can be used but it has some toxicity, limitations, and side effects [9,10]. Thus, there is an urgent need to develop a better treatment method against the VRSA and CRE infections [5,11]. This highlights the emergency of developing alternatives to antibiotics that have high antimicrobial activity with less or negligible toxicity.

Antimicrobial peptides (AMPs) can be an interesting alternative to traditional antibiotics due to its natural occurrence in almost all species, their amphipathic nature, and their assist innate immunity. They have the ability to provide highly effective and non-specific defensive activity against invading pathogens [12,13,14]. Few AMPs have been tested clinically that showed some antimicrobial activity [15]. Though, there are few limitations of these antimicrobial peptides as they can be unstable, interact non-specifically, and have a toxic nature [16]. Some of them are highly effective against bacteria and fungi, and few also impart anticancer activity. The problem with solubility is mostly negligible, and the presence of secondary structures (like helices and beta sheets), with the possibility to modify them, elevate their value for even better treatment. The nanoparticles help in the advancement of nanomedicine, which in turn have helped in targeted drug delivery, low dosage, and high activity with the limitation of toxicity, solubility, and stability [17]. Carbon nanoparticles, like graphene quantum dots, graphene oxide, and CQDs, have received great attraction due to their antimicrobial properties [18]. CQDs are emerging with wide applications in sensors, catalysis, medicine, and bio-imaging. The nanosized CQDs provide advantage with their wide surface area, physiochemical, and optical properties [19]. Their photo-induced redox characteristics and intrinsic optical properties can be enhanced and exploited to introduce a new platform for natural/visible light-activated antimicrobial agents [20]. Thus, they can be used as an antimicrobial agent with the scope to modify their surface charge and functional groups for better performance. They interact with the bacterial surface causing cell wall damage and an increase in endogenous reactive oxygen species (ROS), leading to cytoplasmic leakage and cell death. Studies showed that some CQDs were biocompatible in in vivo and in vitro setup [19,21]. Though they have some limitations, like short excitation/emission wavelength with poor penetration capability, unclear photoluminescence, no antibacterial activity in few cases, and poor multi-color emissions. Consequently, CQDs can be modified and conjugated with peptides to obtain antimicrobial activity. Thus, an eligible alternative to antibiotics are antimicrobial peptides and nanoparticles.

The formation of conjugates using peptides with nanomaterials or other chemicals is possible and provides a wide range to produce new particles with medical applications [17]. HSER (*Homo sapiens* retinoic acid receptor) is a 22-amino acid residue peptide derived from retinoic acid receptor responder protein 2 of *Homo sapiens*. Retinoic acid receptor responder protein 2 was known to exhibit defensive response against gram positive and negative bacteria [22]. The peptide was synthesized and the antibacterial activity against VRSA and *E. coli* was seen to be poor. Furthermore, the carbon quantum dots (CQDs) were synthesized based on our previous studies [23]. Then, conjugation of the peptide was done using CQDs to increase the antibacterial activity of HSER after the formation of HSER–CQDs as a whole.

The present experiment is mainly based on the molecular dynamics (MD) simulation of the designed HSER peptide to understand the structure of the peptide, which was accompanied by the synthesis and characterization of the HSER-CQDs conjugate. Thereafter, the antimicrobial activities of HSER–CQDs were tested against VRSA, and *E. coli* with the determination of their respective MIC (minimum inhibitory concentration). Further, the fluorescence live/dead cell imaging and phase contrast microscope imaging against VRSA and *E. coli* was studied. Thereafter, the mechanism of the HSER–CQDs were studied. Finally, the toxicity tests like hemolytic assay and MTT assay were performed. Thus, the HSER–CQDs showed antibacterial activity that can be used as a better treatment strategy against multidrug-resistant bacterial infections and a potential substitute for antibiotics with medicinal values.

## 2. Experimental

### 2.1. Molecular Dynamics (MD) Simulation

The peptide was derived from the retinoic acid receptor responder protein 2 of *Homo sapiens* due to its defensive response against gram positive and negative bacteria. The structure was constructed directly from sequence (CCVRLEFKLQQTSCRKRDWKKP) using the build structure tool in UCSF Chimera v1.10.2, with standard Φ/Ψ angle values for alpha helix (−57° and −47°, respectively) and Dunbrack rotamer library for side-chains, then hydrogens were added. Molecular dynamics (MD) simulation was prepared and run on GPU (NVIDIA GTX1080Ti) using Gromacs v2018 with Amber99SB force field. The standard leap-frog integrator was used for calculation of trajectories in steps of 2 fs. The Verlet scheme was used for neighbor searching, while the cut-off method was used for Van der Waals interactions at 1.2 nm. Particle mesh Ewald (PME) was used with all electrostatics calculations at 1.2 nm cutoff. Briefly, the peptide was solvated in SPC water model in dodecahedron periodic box and neutralized with Cl^−^ ions. Only 335 steps of steepest descent minimization were required to achieve convergence with maximum force <1000 kJ/mol/nm. NVT equilibration was done for duration of 1 ns using LINCS constraints for H-bonds, and modified Berendsen thermostat coupling (300 K) in two groups (protein and non-protein). NPT equilibration was done for duration of 1 ns using LINCS constraints for H-bonds, modified Berendsen thermostat coupling (300 K), and isotropic Brendsen pressure coupling (1 bar). MD Production was performed in NPT for 1000 ns using LINCS constraints for H-bonds, modified Berendsen thermostat coupling (300 K), and Parrinello-Rahman isotropic pressure coupling (1 bar). Trajectories and energies were saved every 10 ps and the total number of trajectory frames was 100,000. Post MD analysis was done by centering the protein in the box, followed by fitting of backbone and removal of all water molecules from the system. Time evolution of secondary structure was performed according to the standard DSSP method. Visualization and further analysis was done in UCSF Chimera. Clustering analysis of minimal backbone in steps of 50 frames was used to identify the most stable conformation (largest cluster). Root mean square deviations (RMSD) for the backbone (87 atoms) and for all atoms in peptide (191 atoms) were calculated with reference to the first frame (α-helix) and to the representative frame of top cluster.

### 2.2. Chemicals and Synthesis of the HSER-CQDs Conjugate

All the chemicals, reagents for peptide synthesis, CQDs synthesis, and standards were purchased from Sigma-Aldrich (St. Louis, MO, USA) in ACS purity, unless noted otherwise. All the experiments were done maintaining sterile condition to obtain the best results.

According to our previous studies the preparation of the water soluble CQDs were done and briefly the synthesis of CQDs is described [24]. To the solution of citric acid (2.1 g), and Mili-Q water 0.8 mL of ethylenediamine was added in a 100 mL three-necked flask in stirring condition for a couple of minutes (min). Then the volume was made to 50 mL. Then the solution was subjected to microwave irradiation for 10 mins (Multiwave 3000, Anton Paar GmbH, Graz, Austria). The solution was purified by dialysis against Mili-Q with a D-Tube maxi dialyzer overnight to remove the unbound ethylenediamine [23]. Fmoc solid phase synthesis was used to synthesize HSER on Liberty Blue peptide synthesizer (CEM, Matthews, NC, USA). The sequence of HSER is CCVRLEFKLQQTSCRKRDWKKP, a stretch of 22 amino acids. Equal proportion (1:1 ratio) of the HSER and CQDs was added in a vial and kept in rotator Multi Bio RS-24 (Biosan, Riga, Latvia) for 24 h at a constant rotation and time interval vibration. The solution was purified again by dialysis against Mili-Q with a D-Tube maxi dialyzer for 24 h to remove the unbound peptide from the solution.

### 2.3. Characterization of the HSER-CQDs Conjugate

The HSER-CQDs were prepared and visualized under a UV transilluminator at excitation wavelengths (λ_ex_) of 270 and 312 nm (Transilluminator Multiband TFX-35.MC(Vilber Lourmat, Collégien, France). A multifunctional microplate reader Tecan Infinite m200 PRO (Tecan group Ltd., Männedorf, Switzerland) was used to measure the absorbance and fluorescence of CQDs, HSER, and HSER -CQDs [25]. Samples were separated using the KNAUER PLATINblue V6900A HPLC system, which consisted of two P–1 pumps and an auto-sampler AS–1 (Knauer, Berlin, Germany). Mobile phase A and B consisted of acetonitrile 0.1% formic acid and water with 0.1% formic acid. Gradient mode had a flow rate of 0.5 mL/minute. Injected sample volume was 20 µL. Prior to injection, the samples were diluted with mobile phase A to get a final concentration of 10 µg/mL. HPLC was coupled with electrospray-ionization quadrupole time-of-flight mass spectrometer (ESI–qTOF–MS) Bruker Maxis Impact (Bruker Daltonik GmbH, Bremen, Germany). The following parameters of mass spectrometer were used: End plate offset potential, 500 V; capillary potential, 4500 V; nebulizer gas (N2) pressure, 4 bar; drying gas (N2) flow rate, 6 L/min^−1^; drying temperature, 300 °C. Mass range was set from 50 to 3000 *m*/*z*. Positive ion mode was used. Prior to HPLC-MS analysis, a mass spectrometer was calibrated using ESI-TOF tuning mix.

The size distribution and the average size of the nanoparticles were determined using Malvern Zetasizer (NANO-ZS; Malvern Instruments Ltd., Worcestershire, UK), which has quasielastic laser light scattering. The 1.5 mL of the CQDs solution, HSER, and HSER–CQDs were added to the polystyrene latex cell and measured at an detector angle of 173° at 633 nm wavelength, with temperature of 25 °C, the refractive index of 0.30 and real refractive index of 1.59. Fourier transform infrared spectroscopy (FTIR) analysis was done by film of each freeze dried samples using an FTIR spectrophotometer MATTSON 3000 FT-IR (Madison, WI, USA) with a wavenumber range of 400–4000 cm^−1^, with 4 cm^−1^ resolutions.

### 2.4. Cultivation of Bacteria

Bacterial strain, vancomycin-resistant *Staphylococcus aureus* (VRSA) CCM 1767, was obtained from the Czech Collection of Microorganisms, Faculty of Science, Masaryk University, Brno, Czech Republic, and *Escherichia coli* ATCC BAA 2340 was obtained from American Type Culture Collection (ATCC), United States. The media used for the growth of the bacteria was Mueller Hinton (MH) media of 15 mL in Erlenmeyer flasks and the bacteria was inoculated and cultures were kept in shaker at 37 °C at 130 rpm for 24 h. Further, dilution of the culture was done using the MH broth to obtain 0.1 Absorbance (0.5 MacFarland standards) at OD_600 nm_ and used for consecutive experiments [26].

### 2.5. Minimum Inhibitory Concentration (MIC) Determination

The standard broth micro-dilution method (European Committee on Antimicrobial Susceptibility Testing) was used to determine the susceptibility of bacterial cultures against HSER-CQDs and its individual component, with the detection done by the unaided eye. MIC was calculated using the solutions from high to low concentration gradually. The different concentration of HSER–CQDs (250, 125, 75, 50, 25, and 10 µg/mL) was added to the microplate wells and mixed with the bacterial cultures (0.5 MacFarland with final dilution 1:100 using MH medium). Then the plate was incubated at 37 °C for 24 h. The well with lowest concentration of antibacterial agent with almost no bacterial growth was considered the MIC and the control was bacteria without any treatment [26].

### 2.6. Growth Curves and Viability Percentage

The growth curve was obtained using the Multiskan EX (Thermo Fisher Scientific, Waltham, MA, USA) by measuring the absorbance to determine the antibacterial activity. Different concentrations of the HSER–CQDs, HSER, and CQDs were used to check the antibacterial effects on gram positive and negative bacteria. The positive control was only bacteria without any treatment. To compare the activity of HSER–CQDs, the bacteria was treated with different concentration of HSER and CQDs, respectively. Further, the viability percentage was calculated from the absorbance value after 24 h, comparing it with the absorbance value of the positive control [27].

### 2.7. Microscopy of Antibacterial Agents against Bacteria in Ambient Light and Live/Dead Cell Assay

Initially the samples were incubated with bacteria and HSER–CQDs conjugate (respective MIC) for 4 h at 37 °C in shaking incubator. The optical Olympus BX51 fluorescence microscope equipped with a 40× phase contrast lens was used to study the antibacterial activity of the HSER–CQDs against VRSA and *E. coli*. The number of cells observed per sample was observed from 10 randomized microscopic grid fields.

The live/dead cell assay is based on two fluorescent dyes, namely, SYTO9, which permeates both damaged and intact cell membranes, and propidium iodide (PI), to stain only the cells with damaged cell membranes [16]. A total of 1 µL of both the stains were added after the treatment of the bacterial cells for fluorescence microscopy. An inverted Olympus IX 71S8F-3 fluorescence microscope (Olympus Corporation, Tokyo, Japan) equipped with Olympus UIS2 series objective LUCPlanFLN 40× (N.A. 0.6, WD 2.7–4 mm, F.N. 22), a mercury arc lamp X-cite 12 (120 W; Lumen Dynamics, Mississauga, ON, Canada), and a Camera Olympus DP73 was used to obtain the microscopic image to study the outcomes of live/dead bacterial cells assay. The number of cells observed per sample was observed from 10 randomized microscopic grid fields. The images were processed using the Stream Basic 1.7 Software.

### 2.8. Cell Membrane Break and Leakage Assay and Interaction with DNA

Initially, VRSA was treated with HSER–CQDs (25 µg/mL) and incubated at 37 °C for 4 h in shaking condition. Further, the bacterial cell membrane damage and cell cytoplasmic leakage after the treatment by HSER–CQDs was studied by centrifuging the samples, and the supernatant was used as the template for PCR and the amplification was done using 16S rRNA gene primers (16S Forward- ACTGGGATAACTTCGGGAAAC, and 16S reverse- CAGCGCGGATCCATCTATAA), and confirmation was done using agarose gel electrophoresis. The control was the supernatant of VRSA culture without treatment and the 100 bp ladder was used. The gels were visualized and documented under Azure c600 (Azure Biosystems Inc., USA).

Similarly, the genomic DNA (gDNA) from VRSA was isolated and then incubated with HSER–CQDs and kept for 1 h. Then, the samples were used as the template for PCR reaction and the amplification was done using 16S rRNA gene primers, and confirmation was done using agarose gel electrophoresis with respect to the 100 bp ladder. The control was the gDNA of VRSA culture without treatment. The gels were visualized and documented under Azure c600 (Azure Biosystems Inc., Dublin, CA, USA).

### 2.9. Influence and Toxicity of HSER–CQDs on Eukaryotic Cells (Cytotoxicity Assay and Hemolysis Assay)

The PNT1A (prostate epithelial cells), HBL-100 (mammary gland epithelial cells), MDA-MB-468 (mammary gland adenocarcinoma cells), and Du-145 (prostate adenocarcinoma cells) human cell line were cultured and used. The MTT (3-(4,5-dimethylthiazol-2-yl)-2,5-diphenyltetrazolium bromide) assay was used to check the viability. Briefly, in 50 µL medium, the suspension of 5000 cells was added to each well of microtiter plates, followed by incubation for 24 h at 37 °C with 5% CO_2_. After 24 h treatment, 10 µL of MTT (5 mg/mL in phosphate buffered saline (PBS)) was added to the cells and incubated for 4 h at 37 °C. After that, MTT-containing medium was replaced by 100 µL of 99.9% dimethyl sulfoxide (DMSO) and incubated for 5 min, the absorbance of the samples was determined at 570 nm using Infinite m200 PRO (Tecan, Männedorf, Switzerland) [28].

Further, fresh human blood was collected from the volunteer, with signed informed consent, by a venipuncture from an antecubital vein. RBCs were isolated from blood and the suspensions were washed with 150 mM NaCl solution three to five times. RBCs was incubated with the HSER-CQDs for 1 h at 37 °C. The positive and the negative control was 0.1% Triton X-100 and PBS, respectively. The degree of hemolysis was determined by measuring the absorbance of the supernatant at 540 nm, after centrifugation, and calculated according to the following equation:

(1) $$h=\frac{A_{t}-A_{c}}{A_{100\%}-A_{c}}\times 100,$$

where *h* is percentage of hemolysis; *A_c_* is the absorbance of the supernatant from negative control (PBS, pH 7.4); *A_t_* is the absorbance of the supernatant from the samples incubated with the HSER-CQDs; and *A*_100%_ is the absorbance of the supernatant of positive control (0.1% Triton X-100), which causes complete lysis of RBCs [26].

### 2.10. Endogenous Reactive Oxygen Species (ROS) Production Assay

Bacterial endogenous ROS production was measured by using DCFDA Cellular Reactive Oxygen Species Detection Kit (Abcam Ab113851) according to the given protocol and instructions for bacterial cells [29,30]. To the cell suspension of VRSA, 10 µM of 2′,7′–dichlorofluorescein diacetate (DCFDA) was added and kept at 37 °C for 30 min. in the dark condition followed by a wash using 1× washing buffer. Then, VRSA cells were treated with 75 µg/mL of HSER, HSER–CQDs, and CQDs for 3 h. The fluorescence intensity was measured by Tecan infinite m200 pro (wavelength of excitation at 485 nm and emission at 535 nm. The ROS was determined by comparing with the control, which was treated with H_2_O_2_ showing positive ROS production [31].

### 2.11. Microscopic Analysis of HSER-CQDs Interaction against Eukaryotic Cells

The MDA-MB-468 (mammary gland adenocarcinoma cells), Du-145 (prostate adenocarcinoma cells), PNT1A (prostate epithelial cells), and HBL-100 (mammary gland epithelial cells) human cell line were cultured and used. Briefly, the suspension of 5000 cells in 300 µL medium supplemented with growth factors was added to each well of microtiter plates, followed by incubation for 24 h at 37 °C with 5% CO_2_ to ensure cell growth. After 24 h treatment with HSER–CQDs, the cells were observed under an inverted Olympus IX 71S8F-3 fluorescence microscope (Olympus Corporation, Tokyo, Japan) equipped with Olympus UIS2 series objective LUCPlanFLN 40× (N.A. 0.6, WD 2.7–4 mm, F.N. 22), a mercury arc lamp X-cite 12 (120 W; Lumen Dynamics, Mississauga, ON, Canada), and a Camera Olympus DP73 to obtain the bright field microscopic image using Stream Basic 1.7 Software. The number of cells observed per sample was observed from 10 randomized microscopic grid fields.

## 3. Results and Discussion

The synthesis and characterization of HSER–CQDs, along with its antibacterial efficacy against VRSA and *E. coli*, was studied and the MIC was determined. Further the toxicity test showed it has no toxicity against eukaryotic cell lines (prostate and mammary gland epithelial cell lines (HBL-100, and PNT1A). The mechanism of action was based on cell wall damage and leakage of cellular content. Some interaction of the HSER–CQDs with gDNA was observed, and assay showed negligible ROS generation. VRSA and *E. coli* was used as model organism to test the antibacterial effect of the HSER–CQDs due to its pathogenicity [26].

### 3.1. Peptide Structure

**Figure 1:**
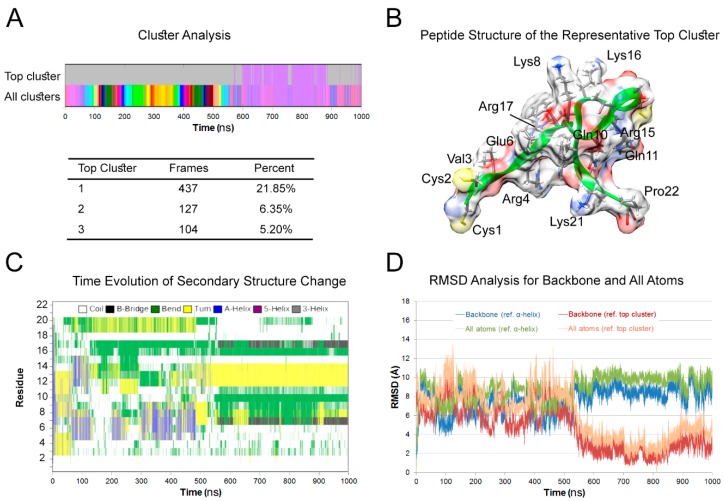
Molecular dynamics simulation of peptide. (**A**) Cluster analysis of Molecular dynamics( MD) trajectories. Snapshots were taken every 50 frames and the final number of frames was 2000. The top cluster was predominant in the second half of the simulation. (**B**) The peptide structure at frame #81,251 is representative of the top cluster. The backbone ribbon is shown in green while the molecular surface is shown in standard heteroatom color. There are 10 intra-model hydrogen bonds in this frame donated by the following atoms: Arg4 (HE, HH21), Gln10 (HE21, HE22), Gln11 (HE21), Arg15 (H, HH11), Arg17 (HE, HH21), and Asp18 (H). Only three of these involved the backbone. (**C**) Time evolution graph of secondary structure change. After the first 550 ns of the simulation, the peptide displayed a stable secondary structure as follows: 1–6 (coil), 7 (β-bridge), 8–11 (bend), 12–14 (turn), 15 (coil), 16 (bend), 17 (β-bridge), 18–22 (coil). (**D**) Root mean square deviations (RMSD) analysis for backbone and all atoms. The structure deviated from the original α-helix in the first frame by range of 5–11 Å. The RMSD for the backbone of the top cluster was below 4 Å, while for all atoms was below 6 Å.

The largest protein conformation cluster was reached after ~550 ns and represented a nearly continuous duration of 21.85% of entire simulation (437 of 2000 frames) (Figure 1A). The representative frame (Figure 1B) for the top cluster was 81,251 (of total 100,000). The video for the last 300 nanoseconds (ns) of simulation, where the peptide has stable conformation, is shown in ESI (Electronic Supplementary Information). The atoms are chloride ions.

In both the average structure of largest cluster and the representative frame, hydrogen bonds were observed rather in the side-chains than the backbone. Namely, Glu6 and Asp18 side-chains provided acceptor oxygens to the amides of arginines and glutamines (Arg4, Arg15, and Arg17 in addition to Gln10 and Gln11). This observation is important for interpretation of FTIR vibrational spectra. Additionally, indicating that possible change in protonation states (due to pH change) will be detrimental for stable folding.

The ime evolution graph of the secondary structure (Figure 1C) shows a combination of alpha helix (residues 5–10) bends and turns in the first half of the simulation. In the second half of the simulation, a stable conformation was formed in the middle of peptide. It was featured by turns (residues 12–14) surrounded by bends and finally a beta bridge connecting residues 7 and 17. The rest of the peptide endings were dominated by random coil (Figure 1C).

RMSD analysis confirmed these observations (Figure 1D). Overall, RMSD were higher by ~1 Å in all atoms compared to backbone-only atoms. With reference to the first frame (α-helix), the deviations in the backbone and all atoms were in the range of 4–11 Å during all simulation and 7–11 Å in the second half. With reference to the representative frame of the top cluster, RMSD dropped from 4–12 Å in the first half to 1–6 Å in the second half of the simulation for both the backbone and all atoms.

### 3.2. Synthesis and Characterization of the HSER-CQD Conjugate

**Figure 2:**
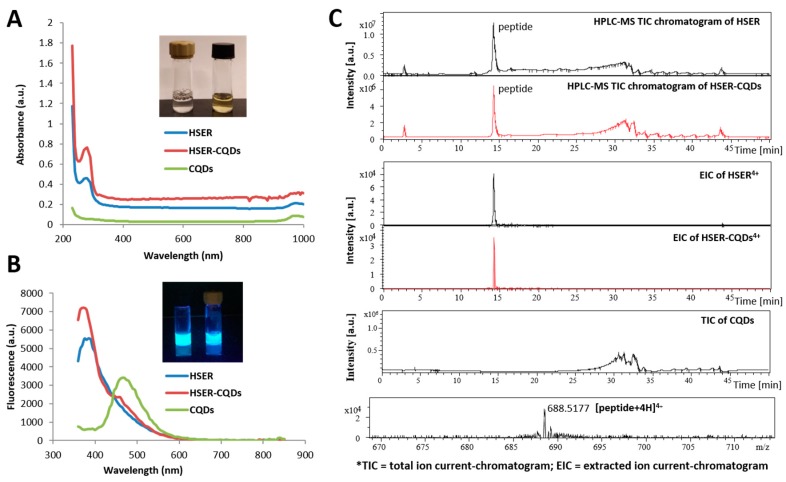
(**A**) The fluorescence spectra were obtained with the excitation of 320 nm and emission at range of 360 to 850 nm; and the bottle with black cap contains Carbon Quantum Dots(CQDs) and the brown-capped bottle contains the HSER-CQDs conjugate showing bluish fluorescence. (**B**) The Absorbance spectra obtained at a range of 230 to 1000 nm. (**C**) Samples were separated on column Zorbax eclipse AAA C18 (3.5 μm particles; 150 × 4.6 mm) using the KNAUER PLATINblue V6900A HPLC system, which consisted of two P 1 pumps and an autosampler AS 1 (Knauer, Berlin, Germany). Mobile phase A consisted of water with +0.1% formic acid. Mobile phase B consisted of acetonitrile with +0.1% formic acid. Gradient mode: 0 min 3% B– > 30 min 97% B– > 40 min 97% B– > 42 min 3% B– > 50 min 3% B– > STOP. Flow rate was 0.5 mL/min. Injected sample volume was 20 µL. Prior to injection the samples were diluted with mobile phase A to get a final concentration 10 µg/mL. HPLC was coupled with ESI-QqTOF mass spectrometer Bruker Maxis Impact (Bruker Daltonik GmbH, Bremen, Germany). The following parameters of mass spectrometer were used: End-plate offset potential, 500 V; capillary potential, 4500 V; nebulizer gas (N_2_) pressure, 4 bar; drying gas (N_2_) flow rate, 6 L/min^−1^; drying temperature, 300 °C. Mass range was set from 50 to 3000 *m*/*z*. Positive ion mode was used. Prior to HPLC-MS analysis a mass spectrometer was calibrated using ESI-TOF tuning mix.

The HSER peptide and CQDs in 1:1 ratio helps in the formation of the HSER–CQDs conjugate, which was dialyzed and observed under UV–transilluminator and showed a bluish fluorescence. The spectrophotometric analysis was done and the absorbance was detected in the spectral range from 230 to 1000 nm and the maxima absorption peak for HSER and the HSER-CQDs conjugate was obtained at 280 nm (Figure 2A). Our experimental results clearly indicate the absence of CQDs absorption peak in visible spectral range. However, the lower absorbance peak of HSER detected at 280 nm belongs to transition of diffused pi electrons of the aromatic ring of tyrosine or tryptophan [32]. On the contrary, the higher absorbance peak of HSER–CQDs was slightly shifted and detected at 270 nm. Such a peak probably belongs to the π–π* transition of aromatic –C=C– and –C–C– bonds in the sp2 hybridized domain of CQDs core [33], which indicates that the interaction between HSER and CQDs has occurred. The fluorescence properties of HSER and CQDs can be observed by two fluorescence peaks at 370 and 445 nm from the respective spectra, as shown in Figure 2B. The CQDs emission peak was found at 445 nm, which is ascribed to surface or molecule center of CQDs [34]. Such fluorescence behavior of CQDs is common and usually is attributed to the diversity of particle size as well as the surface state of CQDs [35]. The lower emission peak of HSER was detected at 370 nm, while the higher emission peak of the HSER–CQDs conjugate was shifted and shows the presence of both peaks at 350 and 425 nm, confirming the interaction between HSER and CQDs. A peak detected at 350 nm usually comes from the presence of carboxyl (–COOH) or amine (–NH2) groups on CQDs surface, which are attributed to n–π* transition of –C=O, C–N or –C–OH bonds in the sp3 hybridized domains [33]. This probably indicates the possible interaction between CQDs surface with carboxyl or amine group from HSER. Xiaohui Gao and co-workers reported that shifts in the emission peak contribute to the strong interaction between water molecules and excited dipole moments in CQDs in solution [36]. Shifting of peaks confirm the structural change in HSER due to interaction with CQDs [37]. The dialyzed HSER CQDs were further characterized using HPLC coupled with ESI–qTOF–MS to obtain the individual peaks for HSER–CQD components, but, due to the small size of CQDs, they were not spotted in HPLC; however, sharp peaks for the presence of HSER were obtained, as shown in the Figure 2C.

**Figure 3:**
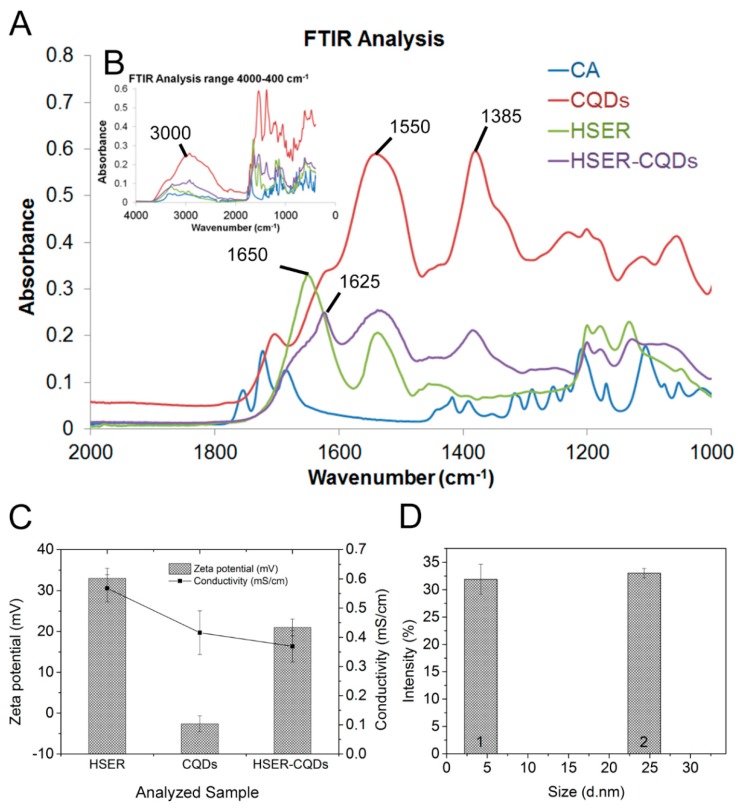
(**A**) FTIR analysis of citric acid (CA), carbon quantum dots (CQDs), peptide (HSER), and peptide-coated CQDs (HSER-CQDs) in the range 2000–1000 cm^−1^. The amide I region shows an increased peak at 1625 cm^−1^ as a result of HSER interaction with CQDs; shouldered with a lower peak at 1650 cm^−1^. The former peak in amides indicates short-range hydrogen bond interactions, while the latter indicates long range hydrogen bond interactions. (**B**) Complete FTIR spectra for the range 4000–400 cm^−1^. A clear and broad band around 3000 cm^−1^ correlates with the hydroxyl group. The overall decrease of CQDs vibrations (red line) confirms successful coating by HSER peptide in the complex HSER-CQDs (purple line). (**C**) The zeta potential of HSER, CQDs and HSER–CQDs, and (**D**) the zeta size of the CQDs is marked as 1 and HSER–CQDs is marked as 2. Data represent the mean ±SD, *n* = 5.

The FTIR analysis (Figure 3A) of the synthesized CQDs showed two major bands in the amide area, namely at 1550 and 1385 cm^−1^, which are characteristic for secondary amines and carboxylates, respectively [38]. The broad range band near 3000s cm^−1^ (Figure 3B) indicates formation of hydrogen-bonded hydroxy groups (–OH) as compared with equivalent CA vibrations [38]. The intensities of these three bands were visibly decreased following the coating by HSER peptide. In peptides and proteins, the amide I band (in the 1600s cm^−1^) is often used as an indicator of secondary structure. This is based on the contributions of C=O stretching and C-N stretching, which are influenced by the strengths of hydrogen bonds involving them [39]. Accordingly, the α-helix structure is correlated with frequency range 1660–1648 cm^−1^, whereas the β-sheet structure is correlated with frequency range 1640–1625 cm^−1^. Fortunately, due to available data from MD simulations, it is clear that such interpretation of structure can be biased in this situation. Predicted HSER structure appeared to be stabilized by amide-based electrostatic interactions of side-chains (Hydrogen bonds). In a more precise description, the vibrations of the backbone do not involve many hydrogen bonds and thus they can be assigned to the disordered secondary structure with frequency range 1657–1642 cm^−1^ [40]. The 1625 cm^−1^ in HSER–CQDs is an indicator of short range backbone aggregation, while the decrease in 1650 cm^−1^ correlates with loss of random backbone and interrupted side-chain interactions in HSER–CQDs. The contribution of amide bands is dominated by the backbone peptide bond, nevertheless, several side-chain vibrations fall in the amide I and II range [41]. Their extension coefficient (M^−1^cm^−1^) contributions in the amide I are in the following range: Arg (ε = 300–490), Gln (ε = 360–380), and Lys (ε = 60–130). As for the amide II region (in the 1500s cm^−1^) they are in the following range: Gln (ε = 220–240), Asp (ε = 290–380), Glu (ε = 450–470) and Lys (ε = 60–130). Furthermore, the Cys thiol (–SH) stretching peak at 2551 cm^−1^ [41] was absent in both HSER and HSER–CQDs spectra, suggesting that sulfide bridges were formed in both cases. Overall, FTIR vibrational spectra confirmed the coating of CQDs with HSER peptide, mostly via strong non-covalent aggregation. This was characterized by amide-based short-range hydrogen bonds (1625 cm^−1^) between peptide backbone and secondary amines, carboxylates, and hydroxyl groups of the CQDs surface.

However, the particle size distribution showing average particle size and the zeta potential of the prepared HSER–CQDs were analyzed using DLS after purification, using dialysis in D-Tube maxi dialyzer tubes against Mili-Q (Figure 3C,D). Thereafter, the formation of nanoparticles was also confirmed by zeta size and potential measuring. Zeta size results obtained indicates that the size of CQDs is 4 ± 2 nm, while the size of HSER-CQDs was found to be 23 ± 3 nm (Figure 3D). Furthermore, the value of the zeta potential showed that the lowest zeta potential was found in case of CQDs at −3 mV, and the highest zeta potential show HSER with 33 mV. Finally, the HSER-CQDs conjugate reveal the change of zeta potential after interaction to be 23 mV, showing the tendencies to aggregate which can be connected with peptide aggregation on the surface of nanoparticles. Thus, after the characterization of HSER–CQDs was completed, it was used to further study the antibacterial activity against pathogenic bacterial strains.

### 3.3. Effect of HSER-CQDs on Different Bacterial Strains

The antibacterial activity of HSER-CQDs against VRSA and *E. coli* was initiated by determining the MIC using broth micro-dilution method. The MIC value of HSER-CQD against VRSA and *E. coli* was seen to be 25 and 50 µg/mL, respectively, and the concentrations below showed turbid solutions proving the presence of the bacteria, whereas the HSER and CQDs were found to have no prominent antibacterial effects or a higher concentration was needed to be applied (>125 µg/mL) (shown in Table 1).

**Figure 4:**
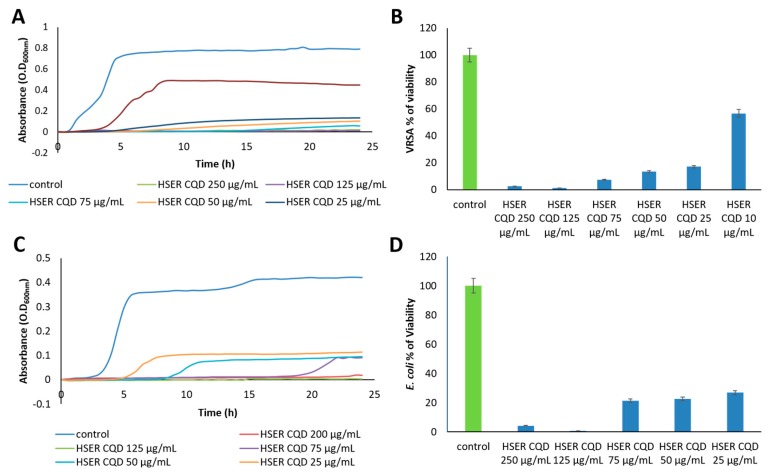
(**A**) The growth curve analysis of VRSA, (**B**) the viability percentage of VRSA cells, (**C**) the growth curve analysis of *E. coli,* and (**D**) the viability percentage of *E. coli*. Data represent the mean ±SD, *n* = 5. VRSA = Vancomycin Resistant *Staphylococcus aureus*.

Thereafter, the growth curve analysis was performed to understand the stages of the growth of VRSA and *E. coli* for 24 h in the presence of the HSER-CQDs. The 24 h growth curve showed the concentration from 75 µg/mL and below, showed a rise in viability of *E. coli* cells, as seen in Figure 4C, but the inhibition was still more than 70%, whereas in case of VRSA the concentration below 25 µg/mL showed very low antibacterial activity, as shown in Figure 4A. Thus, the constant inhibitory effects of HSER–CQD for 24 h against VRSA was observed with the less concentration of 25 µg/mL, but in case of *E. coli* the concentration of 75 µg/mL showed prominent inhibitory effects compared to lower concentrations. Whereas, individually HSER and CQDs did not show any sign of prominent inhibition against VRSA and *E. coli,* as shown in Figures S1 and S2. Thus, the HSER–CQDs showed prominent inhibitory effects and high antibacterial activity compared to the individual components.

Lastly, the percentage of viability after 24 h treatment with HSER–CQDs against VRSA for concentration 125 µg/mL was seen to be less than 1.5%, but lower concentrations, like 75, 50, and 25 µg/mL, showed viability below 18%. Further decrease in HSER–CQDs concentration had no such high inhibitory effects (10 µg/mL (>57%)), as shown in Figure 4B. In case of *E. coli*, the percentage of viability was below 1% for 125 µg/mL, but for lower concentration, like 75, 50, and 25 µg/mL, showed viability below 27%, as shown in Figure 4D. Therefore, viability percentage of inhibition for VRSA was prominent until 25 µg/mL, with the inhibition of more than 82%, but in case of *E. coli*, inhibition was best at 125 µg/mL (>99%), but at 25 µg/mL the inhibition was more than 73%. Whereas, the individual components of HSER showed no prominent activity against VRSA, with an inhibition of 32.32% at 125 µg/mL, and in case of *E. coli* the inhibition was around 22.23% at 125 µg/mL, but CQDs too showed no prominent antibacterial activity against VRSA (32.67%) and *E. coli* at 125 µg/mL (Supplementary Figures S1 and S2). Thus, the HSER–CQDs work better as antibacterial agents against the VRSA and *E. coli* in lower concentrations compared to the individual components.

### 3.4. Microscopic Estimation of Live/Dead Cells and Damage of DNA (Bacterial Cells)

**Figure 5:**
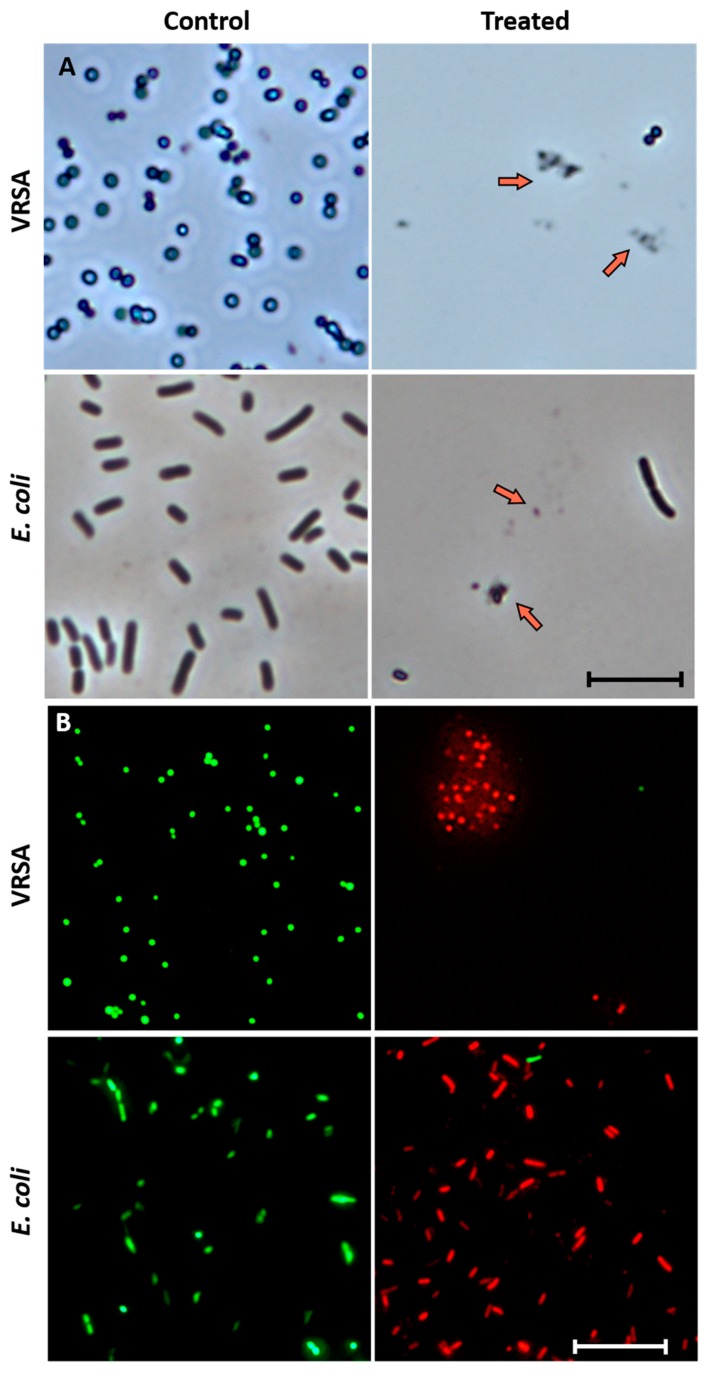
(**A**) The phase contrast microscopic images of VRSA and *E. coli,* with orange arrows showing the dead cells with cell debris. Scale is 5 µm. (**B**) The live/dead cell images of VRSA and *E. coli,* where cells stained with SYTO9 are live cells with green fluorescence and cells stained with PI are dead cells with red fluorescence. Scale is 10 µm. VRSA = Vancomycin Resistant *Staphylococcus aureus*.

The phase contrast condition in the optical Olympus BX51 fluorescence microscope was used for further confirmation about the antibacterial activity of the HSER–CQDs against VRSA and *E. coli*. Decrease in the viable bacterial cell numbers and disruption of the cell with loss of cell integrity due to rupture of the cell wall cause a huge change in bacterial morphology, cytoplasmic leakage, and the presence of cell debris, which were seen under the microscope for both VRSA and *E. coli* after the treatment with HSER–CQDs, whereas the control bacterial samples with no treatments showed high number of viable cells with full cell integrity, no change in morphology, and absence of cell debris, as shown in Figure 5A.

Furthermore, to validate the results, the viability of the VRSA and *E. coli* after treatment with HSER-CQDs was observed by the live/dead assay using an inverted fluorescence microscopy. The bacterial samples treated with the HSER-CQDs showed a considerable decrease in the viability of the bacterial cells, showing almost negligible numbers of green fluorescent cells (SYTO9 stained), but a significant increase in number of dead cells with red fluorescence (PI stained). On the contrary, in case of controls cells, the number of live green fluorescent cells were high with almost no dead cells were observed, as shown in Figure 5B. Similarly, optical microscopic image (as shown in Figure S4) further validates the antimicrobial activity of the HSER-CQDs against the VRSA and *E. coli*. 

Thus, the results showed prominent reduction in the viability of bacterial cells due to the presence of HSER-CQDs causing cell death by disrupting the cell wall, causing loss of cell morphology and cell death, proving its high-antimicrobial activity against both bacterial strains [42]. Thus, the probable mechanism of action for HSER-CQDs can be the cell wall disruption of bacterial cells.

### 3.5. Mechanism of Action by Interaction with Bacterial Cells, DNA, Changes in Secondary Structure, and Membrane Insertion

**Figure 6:**
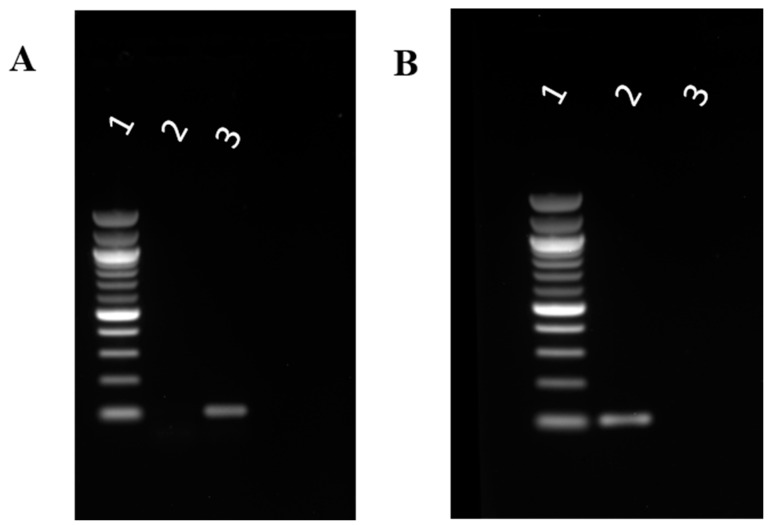
(**A**) Cell wall damage and leakage assay, where (1) 100 bp ladder, (2) PCR product of 16S rRNA of supernatant of VRSA cells without any treatment, and (3) PCR product of 16S rRNA of supernatant of VRSA cells treated with HSER–CQDs. (**B**) Interaction of the HSER-CQD with gDNA was studied using gel electrophoresis; (1) the 100 bp ladder, (2) PCR product of 16S rRNA of VRSA genomic DNA (control), and (3) PCR product of 16S rRNA of VRSA genomic DNA treated with HSER–CQDs. VRSA = Vancomycin Resistant *Staphylococcus aureus*.

The previous studies under microscope showed the loss in cell integrity and disruption of the cells. To further confirm the interaction of HSER–CQDs with the bacterial cell wall causing disruption and leakage of the cellular content, the HSER–CQDs were treated with VRSA cells, and the supernatant after 4 h of treatment was used to study the presence of DNA. The treated VRSA cells supernatant on PCR amplification, using the 16S rRNA primers, confirmed the presence of the band of DNA at 106 bp, whereas the control without any treatment showed no band, as shown in Figure 6A. Thus, it can be concluded that the HSER–CQDs treated cell samples were indeed having cell wall damage and leakage of DNA. Further, the interaction of HSER–CQDs in presence of gDNA was performed separately. Then the PCR amplification of gDNA after treatment with HSER–CQDs for 16S rRNA showed no band, but for the control (gDNA without treatment) a prominent band at 106 bp was obtained, as shown in Figure 6B. Thus, it was seen that the HSER-CQDs not only interacted with the cell wall, which caused the cell disruption and leakage, but also interact with gDNA when treated separately and prevents its PCR amplification or replication. Therefore, the HSER–CQDs interacts with the DNA when incubated separately, but when they are treated with the cells they interact with the cell wall and no strong interactions can happen with the total DNA content, as a result of which we saw a prominent band from the PCR amplification using the supernatant after the treatment.

Based on net charge, cell-penetrating peptides can be either hydrophobic, amphipathic, or cationic [43]. The secondary structure of many cell-penetrating peptides and antimicrobial peptides has been studied in connection to their interaction with membranes. Some peptides, like penetratin, adopt either α-helix or β-sheet structure when interacting with the membrane, while polycationic peptides (like Tat and short poly-arginines) remain in unstructured random coils [44]. These peptides have high affinity to membranes with high concentrations of anionic lipids, and require formation of salt-bridges to obtain favorable energetic values for insertion in the membrane [45]. The pH (Low) Insertion Peptides (pHLIP peptides (low pH-dependent)) were shown to transform between three states, from unstructured highly-soluble to α-helix transmembrane state [46]. The naturally-occurring SMAP-29 peptide was shown to exhibit different antibacterial activities associated with its orientation when it was immobilized on magnetic beads [47]. Novicidin is an example of peptides that form a stabilized helix inside the lipid membrane [48]. A study on Novicidin showed that the binding process is ruled by membrane partitioning (how much membrane surface area per peptide), not only by helicity or charge [49].

We have showed in this work that the HSER peptide adapted a loop-shaped structure in MD simulation, and displayed random secondary structure signature by FTIR analysis (that can be interpreted as α-helix in other cases). Following the immobilization of HSER on CQDs, β-sheet structure signature was detected, which also correlates with short-range inter-chain interactions. The data clearly shows that the immobilized peptide backbone structure is different from the free one. Based on previous insights, this change in secondary structure can explain the change in peptide antimicrobial activity. The β-sheet structure realigns the peptide sequence in an alternating and amphipathic way that can break some of the electrostatic strains in the side-chains; allowing them to directly interact with the membrane. Lastly, the ROS generation against the bacterial cells was seen using DCFDA probe and spectrophotometer. The results showed in Figure S3 that the cells treated with HSER–CQDs showed negligible ROS generations in comparison to the positive control treated with H_2_O_2_. The HSER and CQDs treated cells showed higher ROS generations in comparison to HSER–CQDs-treated cells.

**Figure 7:**
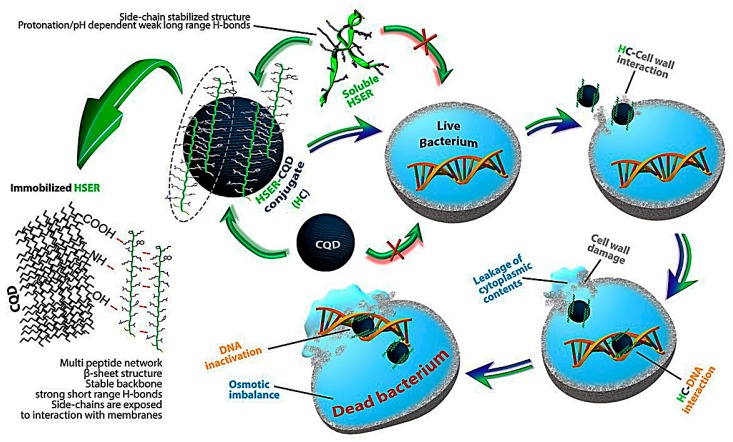
The mechanism of action of HSER–CQD, due to change in the structure of the peptide after interacting with CQDs, causing cell wall interaction, leads to leakage of cytoplasmic content and interaction with DNA, causing its inactivation.

Finally, the mechanism of interaction for HSER–CQDs is based on the change of structure of the HSER after coating on CQDs surface, which causes cell wall interaction leading to cell wall disruption, with loss of cell integrity causing the leakage of intercellular component with no ROS generations, as shown in Figure 7; but when incubated separately with gDNA it prevents it from amplification.

### 3.6. Influence and Cytotoxicity Test of HSER–CQD on Eukaryotic Cells

The cytotoxicity of HSER–CQDs was estimated using the MTT assay PNT1A (prostate epithelial cells), Du–145(prostate adenocarcinoma cells), MDA MB 468 (mammary gland adenocarcinoma cells), and HBL–100 (mammary gland epithelial cells). MTT assay is based on reduction of MTT by succinate dehydrogenase reflecting metabolic healthy conditions of cells [50,51]. The viability was studied using different concentrations of HSER–CQDs, as shown in Figure S5A–S5D. In case of normal prostrate epithelial cells (PNT1A), the CQDs did not show any sign of toxicity at any concentration tested. However, HSER–CQDs incubated with normal mammary gland epithelial cells (HBL–100) at concentration of 125 μg/mL showed reduction in viability of cells by 26%, but lower concentration showed no toxicity at all. Furthermore, the mammary adenocarcinoma cells showed prominent reduction in viability until 31.25 µg/mL, whereas prostate adenocarcinoma cells showed prominent reduction in viability in different concentrations of the HSER–CQDs conjugate. Thus, cytotoxicity was seen against adenocarcinoma cells, but no prominent cytotoxicity was seen against the normal epithelial cell lines in the presence of HSER–CQDs.

Further, the hemolytic effects of the HSER–CQDs at different concentrations was studied using blood with positive control as 0.1% Triton–X (100% hemolysis) and negative control as PBS (no hemolysis). The 75 and 50 μg/mL concentrations of the HSER–CQDs conjugate showed negligible hemolysis, almost below 1.5% of hemolysis, as shown in Figure S5E. Thus, the concentration at which the conjugate works showed no toxicity towards red blood cells.

**Figure 8:**
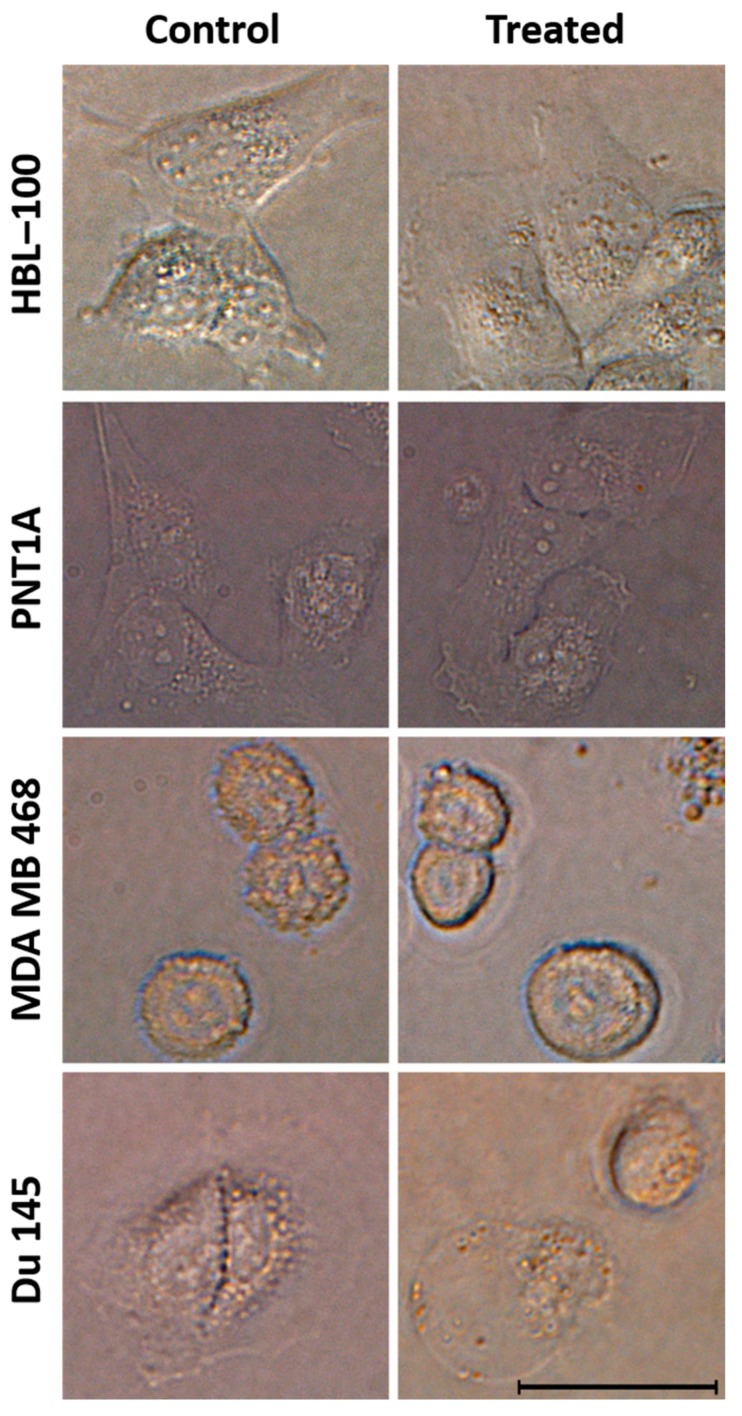
The microscopic image of four types of human cell lines (HBL–100, PNT1A, MDA MB 468, and Du–145) after treatment using HSER–CQDs. Scale is 20 µm.

Finally, the cytotoxicity was confirmed by microscopic observation under an inverted microscopy. The results after treatment of HBL–100, PNT1A, and MDA MB 468 using HSER–CQDs showed no morphological changes in comparison to the control cells. Whereas in case of Du–145, the treated cells, when compared to control cells, showed considerable change in the morphology of few cells with loss of cell integrity and cellular leakage (shown in Figure 8). Thus, the results showing the high viability of normal cells with some toxic activity towards Du-145 adenocarcinoma cells in presence of HSER–CQDs proved that they are biocompatible and non-toxic towards normal human cells in vivo.

## 4. Conclusions

In the present study, HSER was designed from retinoic acid receptor responder protein 2 of *Homo sapiens* due to its defensive response against gram positive and gram negative bacteria. The structural analysis of HSER was computationally done using MD simulation and synthesis was performed. But the peptide showed no prominent antibacterial activity against VRSA and *E. coli*. Thereafter, the synthesis of the CQDs was done based on our previous work and conjugated with HSER to increase its activity. The characterization was done using spectrophotometer, HPLC coupled with ESI–qTOF mass spectrometer, UV-transilluminator, zeta potential and size, and FTIR. The prominent antibacterial activity was seen against VRSA and carbapenem-resistant *E. coli* using growth curve analysis and viability percentage, along with the determination of MIC against respective bacteria. The antibacterial activity was further confirmed by microscopic analysis, showing loss of cell integrity, leakage of cellular content due to cell disruption, and with cellular debris. The live/dead cells assay showed the presence of a high amount of dead bacterial cells. The mechanism of action is based on change in structure of HSER after interacting with the CQDs, which was supported by FTIR results showing that the cell wall disruption occurred along with some interaction in presence of gDNA, which stop its amplification. Finally, the HSER–CQDs were found to be compatible with RBCs and nontoxic against normal human epithelial cells, with the help of MTT and hemolytic assay and further confirmed by microscopic analysis. Thus, HSER–CQDs can be used as a substitute to antibiotics.

## Figures and Tables

**Table 1:** Determining the Minimum Inhibitory Concentration (MICs) of HSER-CQD by broth micro-dilution method.

Bacterial Strains	HSER-CQDs (μg/mL)	HSER (μg/mL)	CQDs (μg/mL)
**VRSA**	25	>125	>125
***E. coli***	50	>125	>125

## Supplementary Materials

The following are available online at https://www.mdpi.com/2079-4991/10/2/325/s1, Figure S1: Growth curve and viability percentage in presence of HSER, Figure S2: Growth curve and viability percentage in presence of CQDs, Figure S3: ROS, Figure S4: Optical image of VRSA and *E. coli*, Figure S5: The cytotoxicity test.
