# Supplementary material for: Peptide-Carbon Quantum Dots conjugate, Derived from Human Retinoic Acid Receptor Responder Protein 2, against Antibiotic-Resistant Gram Positive and Gram Negative Pathogenic Bacteria
Source: Nanomaterials (Basel). 2020 Feb 14;10(2):325. doi: 10.3390/nano10020325 (PMC7075150; doi:10.3390/nano10020325)
Supplement: Supplementary file 1 [file nanomaterials-10-00325-s001.pdf]

Supplementary Data for:

Carbon Quantum Dot – Peptide conjugate, derived from Human Retinoic Acid Receptor Responder protein 2, against antibiotic resistant gram positive and negative pathogenic bacteria

Aninda Mazumdar[1,2]*, Yazan Haddad[1,2], Vedran Milosavljevic[1,2], Hana Michalkova[1], Roman Guran[1,2], Sukanya Bhowmick[1,2], Amitava Moulick[1,2]*

[1]*Department of Chemistry and Biochemistry, Mendel University in Brno, Zemedelska 1, CZ-613 00 Brno, Czech Republic*

[2]*Central European Institute of Technology, Brno University of Technology, Purkynova 123, CZ-612 00 Brno, Czech Republic*

***Corresponding author**

Aninda Mazumdar, Department of Chemistry and Biochemistry, Mendel University in Brno, Zemedelska 1, CZ-613 00 Brno, Czech Republic; E-mail: anindamazumdar@gmail.com, xmazumda@mendelu.cz; phone: +420545133350

Amitava Moulick, Department of Chemistry and Biochemistry, Mendel University in Brno, Zemedelska 1, CZ-613 00 Brno, Czech Republic; E-mail: amitavamoulick@gmail.com; phone: +420545133350

1. Growth curves and viability percentage of the individual component

Growth curve and viability percentage of HSER against VRSA and *E. coli*. The figure S1 showed the effects of HSER against the bacteria is not so promising and the viability of the bacteria is quite high.

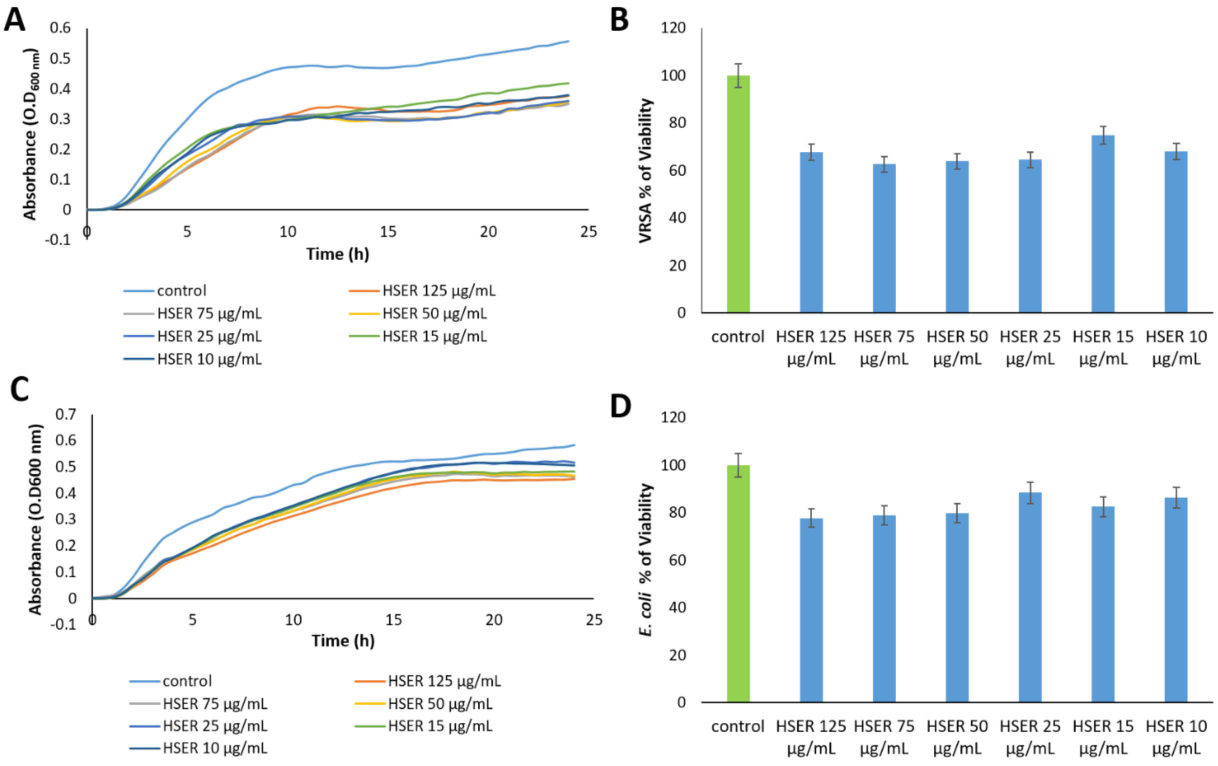

Figure S1: Growth curve and viability percentage of HSER against VRSA (A and B) and E. coli (C and D).

Growth curve and viability percentage for CQDs against VRSA and *E. coli*. The figure S2 showing the effects of CQDs is not prominent and the viability of the bacteria are high even after treatment.

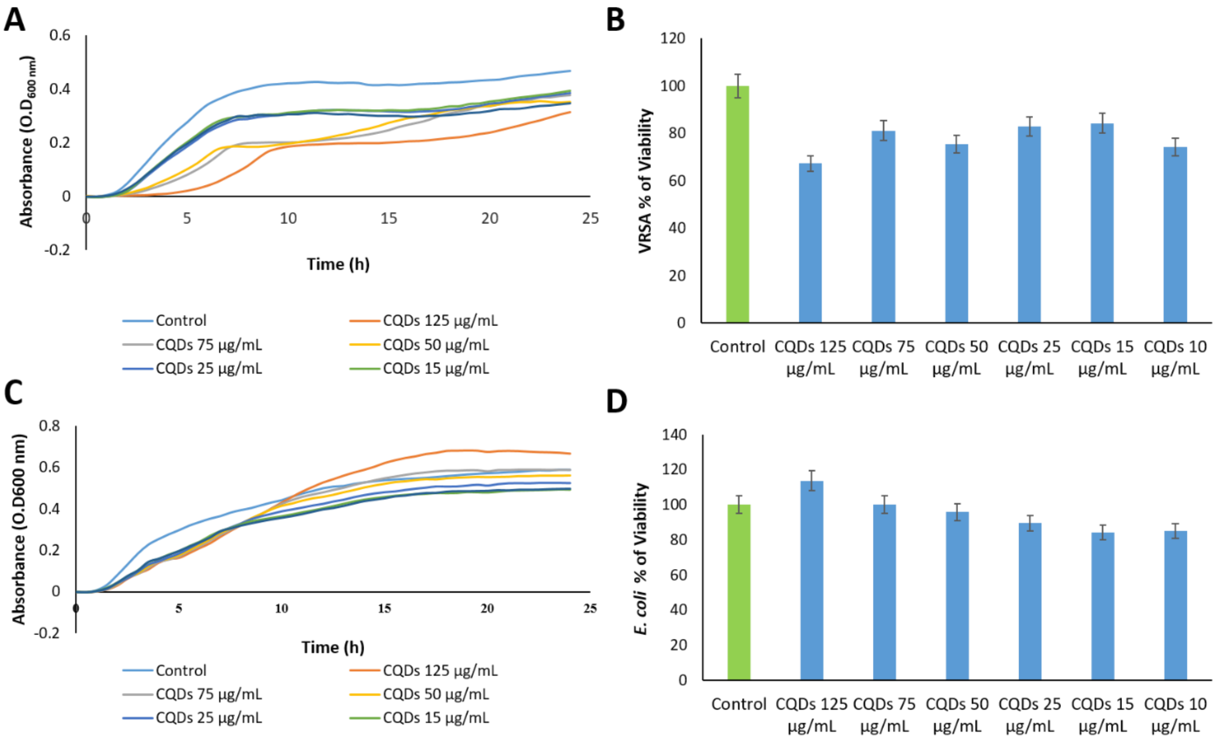

Figure S2: Growth curve and viability percentage of CQDs against VRSA (A and B) and E. coli (C and D).

2. Polymerase chain reaction (PCR)

The primer sequence for forward primer is 16s F- ACTGGGATAACTTCGGGAAAC, and reverse primer 16s R- CAGCGCGGATCCATCTATAA. Primers were obtained from Sigma Aldrich, United Kingdom. Pre-stained Master mix were obtained from New England Biolab S M0486S.

The reaction mixture contains 12.5 µL of the master mix, 1 µL of 400 nM of each primer, the 4 µL of 34 ng template DNA or treated DNA or supernatant of the treated samples were added and finally 6.5 µL of nuclease free water was added to make 25 µL total reaction volume. The PCR was run annealing temperature was 58 °C and 30 cycle extension was done.

3. Reactive Oxygen Species (ROS) assay

The ROS assay of HSER–CQDs, HSER, and CQDs. The HSER–CQs showed the lowest reactive oxygen species generation compared to HSER and CQDs.

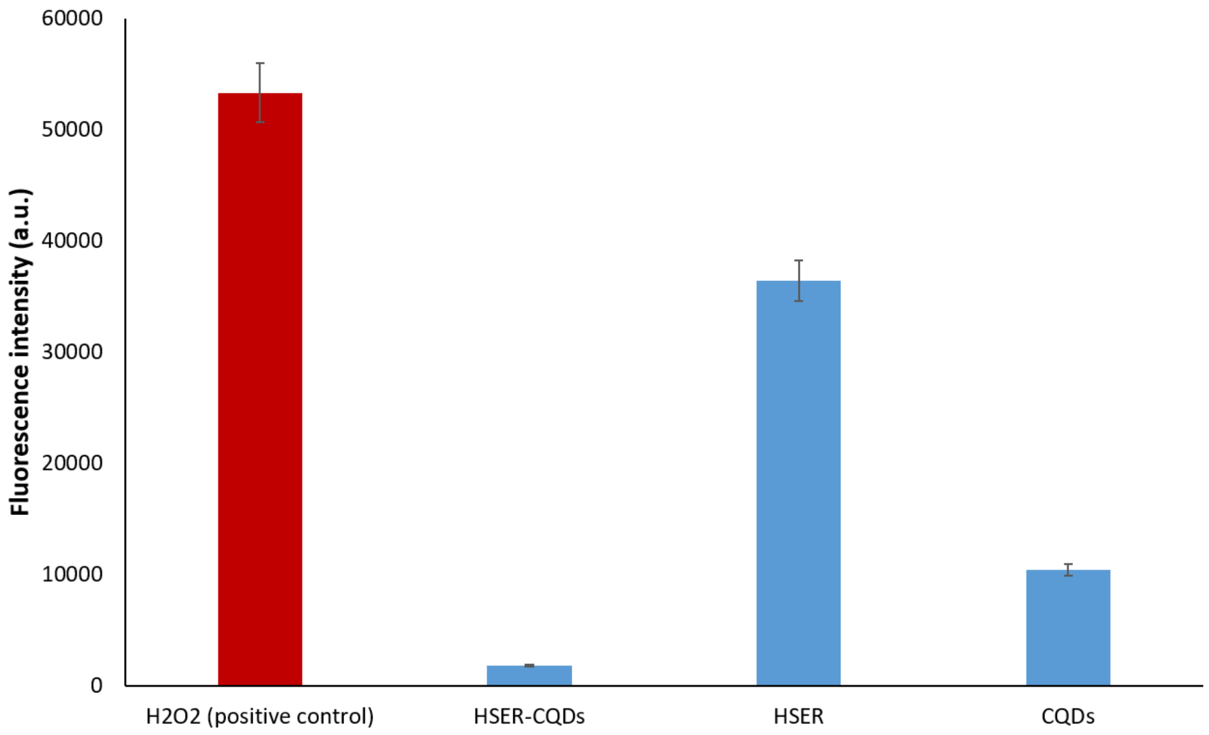

Figure S3: Reactive Oxygen Species (ROS) assay using HSER–CQDs, HSER, CQDs and Positive control. Data represent the mean ±SD, n=5.

4. <u>Bright field microscopy</u>

Optical microscopic image of VRSA and *E. coli* control and HSER–CQDs treated cells.

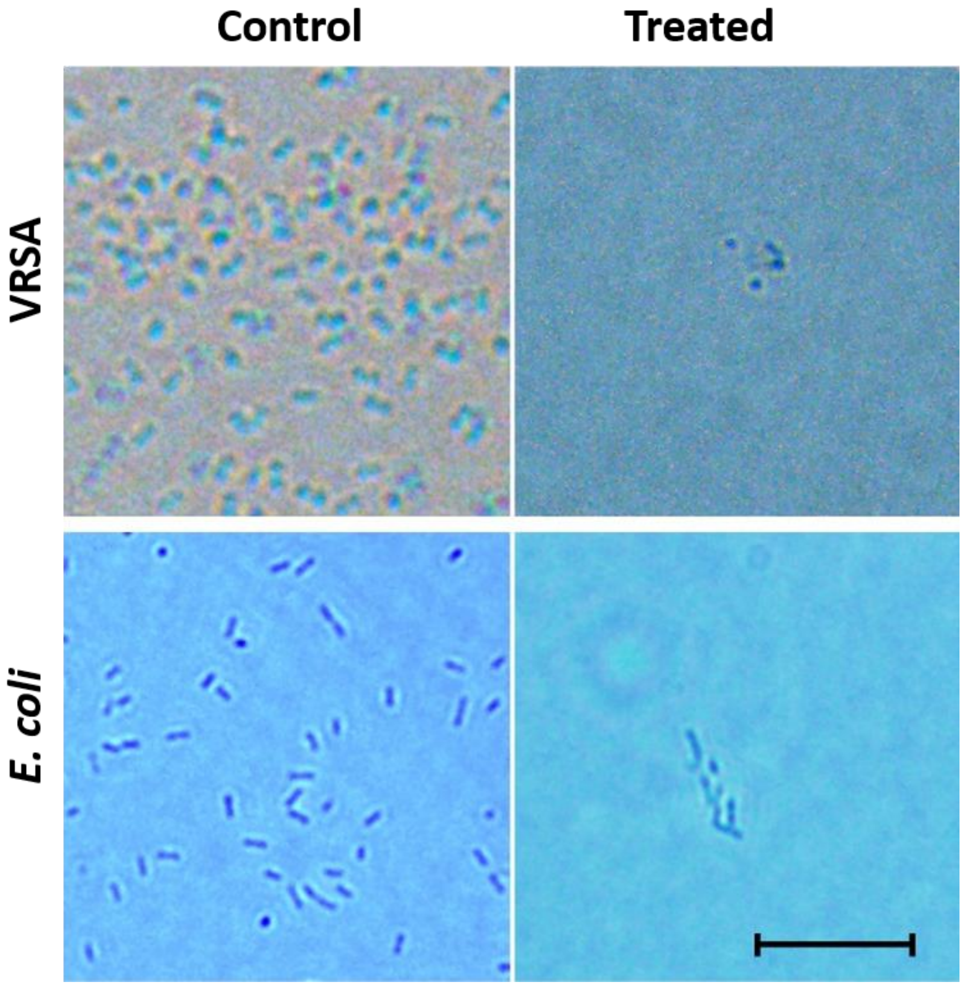

Figure S4: The optical images of VRSA and E. coli with treatment showed cell rupture and cell debris but no debris was seen in the control. Scale is 10 μm.

5. *Cytotoxicity assay*

The cytotoxic test of HSER–CQDs using MTT assay and hemolytic assay against eukaryotic cells. Showing the HSER–CQDs are not toxic.

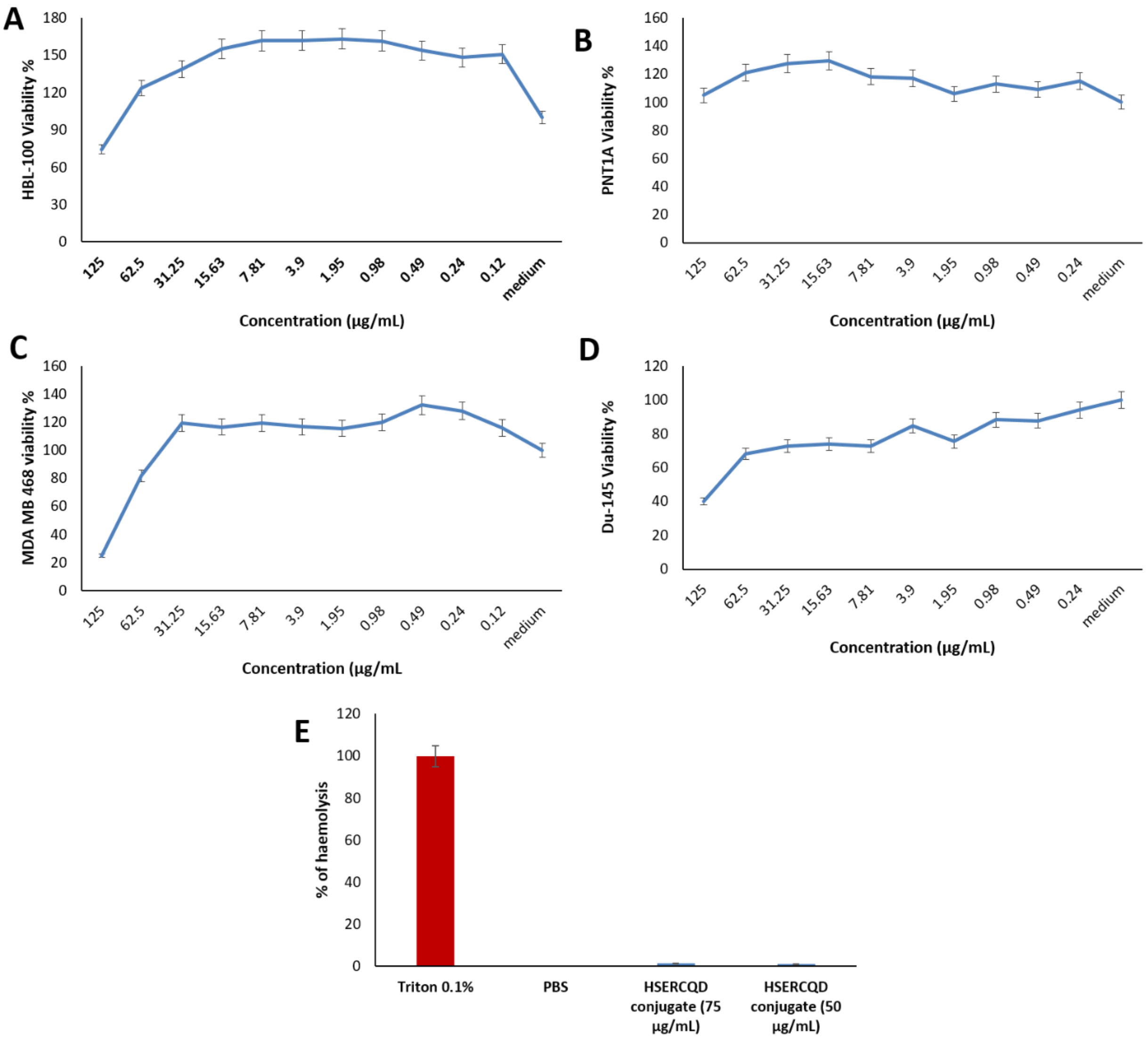

Figure S5: The cytotoxicity test was done using HSER-CQD in different concentrations against four types of human cell lines (MTT assay) and RBCs, A) Viability percentage of HBL – 100 (mammary gland epithelial cells), B) Viability percentage of PNT1A (prostate epithelial cells), C) Viability percentage of MDA-MB-468 (mammary gland adenocarcinoma cells), D) Viability percentage of Du-145 (prostate adenocarcinoma cells), and E) The percentage of haemolysis. Data represent the mean ±SD, n=5.
